# Late Arterial Switch Surgery Under ECMO Support in a Patient with Transposition of the Great Arteries with Intact Ventricular Septum: a Case Report

**DOI:** 10.21470/1678-9741-2019-0106

**Published:** 2020

**Authors:** Sertac Haydin, Erkut Ozturk, Okan Yildiz, Behzat Tuzun, Alper Guzeltas

**Affiliations:** 1 Department of Pediatric Cardiovascular Surgery, Saglik Bilimleri University, Istanbul Mehmet Akif Ersoy Thoracic and Cardiovascular Surgery Training and Research Center Hospital, Istanbul, Turkey; 2 Department of Pediatric Cardiology, Saglik Bilimleri University, Istanbul Mehmet Akif Ersoy Thoracic and Cardiovascular Surgery Training and Research Center Hospital, Istanbul, Turkey.

**Keywords:** Transposition of the Great Vessels, Arterial Switch Operation, Ventricular Septum, Extracorporeal Membrane Oxygenation, Angiography

## Abstract

A 30-month-old male patient with transposition of the great arteries with intact ventricular septum (TGA/IVS) is presented. Arterial switch operation (ASO) was performed in the light of echocardiographic and angiographic findings. The patient remained under extracorporeal membrane oxygenation support for seven days postoperatively, and his cardiac functions returned to normal at the postoperative 10^th^ day. He was discharged at the postoperative 20^th^ day. The present case, which presents one of the most advanced ages at operation for TGA/IVS among previously reported cases, is used to discuss late ASO in this study.

**Table:** 

Abbreviations, acronyms & symbols				
ACC	= Aristotle Comprehensive Complexity		LVMI	= Left ventricular mass index
ACT	= Activated clotting time		PA	= Pulmonary artery
ASO	= Arterial switch operation		PEEP	= Positive end-expiratory pressure
BNP	= B-type natriuretic peptide		PIP	= Peak inspiratory pressure
CPB	= Cardiopulmonary bypass		RA	= Right atrium
ECG	= Electrocardiography		RACHS-1	= Risk Adjustment in Congenital Heart Surgery
ECLS	= Extracorporeal life support		RV	= Right ventricle
ECMO	= Extracorporeal membrane oxygenation		SO_2_	= Oxygen saturation
FiO_2_	= Fraction inspired oxygen		TGA	= The great arteries
IVS	= Intact ventricular septum		TPS	= Technical Performance Score
LA	= Left atrium		VSD	= Ventricular septal defect
LV	= Left ventricle			

## INTRODUCTION

Primary arterial switch operation (ASO) has become the standard treatment of choice for transposition of the great arteries with intact ventricular septum (TGA/IVS) in the first few weeks of life^[1]^. However, there is no standard treatment protocol in patients older than three weeks of age. Different centers perform staged ASO or the Senning procedure^[2,3]^. Recently, successful arterial switch procedures were reported in elderly patients with the aid of technological improvements and advanced intensive care support^[4,5]^. In this report, a successful arterial switch procedure with extracorporeal membrane oxygenation (ECMO) support in a patient with TGA/IVS is presented.

## CASE REPORT

**Fig. 1:**
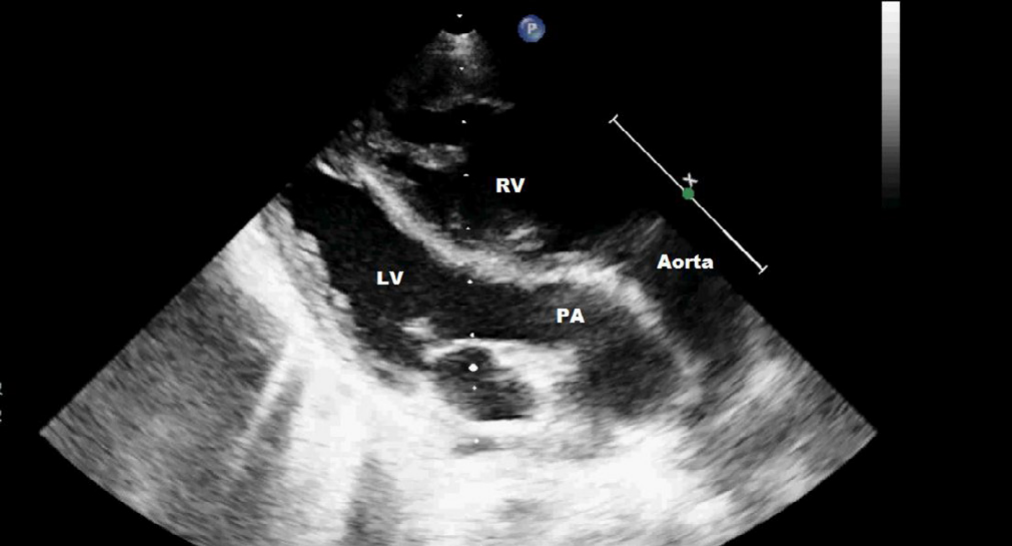
Parasternal long axis transthoracic echocardiography. LV=left ventricle; PA=pulmonary artery; RV=right ventricle

A 30-month-old male with a body weight of 12 kg was admitted to our clinic. There was no other pathology in his physical examination except central cyanosis (72% with pulse oximetry). Normal sinus rhythm with a right axis and right ventricular (RV) dominance were present in the electrocardiographic evaluation. Transthoracic echocardiography demonstrated TGA and an atrial septal defect of 10 mm in diameter with color Doppler ultrasonography. There was no left ventricular (LV) systolic dysfunction or outflow obstruction (Figure 1). There was a banana-shaped LV involution. The LV end-diastolic posterior wall thickness was 3 mm (z score: -1.59), end-diastolic interventricular septum thickness was 6 mm (*z* score: 0.6), end-diastolic LV diameter was 20 mm (*z* score: -4), and LV mass index (LVMI) was 24.7 gram/m^2^ (*z* score: -2.7). The measured LV/RV pressure ratio was 0.5 at the angiography. It was decided to proceed with an arterial switch procedure.

The operation was conducted with cardiopulmonary bypass (CPB) through aortic and bicaval cannulation. Mild hypothermia and del Nido cardioplegia were utilized. Following the preparation of coronaries as small buttons, the LeCompte maneuver was done and ascending aorta was reconstructed via an end-to-end anastomosis. Then, the aortic cross-clamp was released, allowing the neo-aortic root to distend. The ideal locations for the coronary arteries on the neo-aorta were marked with a sterile pen. Stab wounds were made at these marks, taking care not to injure the previously marked anterior neo-aortic commissure, which was marked with a Prolene® suture. Following this, the aortic cross-clamp was reapplied. The location of the anterior neo-aortic commissure was confirmed through the holes, and the openings were enlarged to accommodate the coronary buttons. Pulmonary artery reconstruction was performed after the removal of the cross-clamp with a single autologous pericardial patch that had been treated with glutaraldehyde for three minutes. Ultrafiltration during bypass and modified ultrafiltration after bypass were used. A left atrial pressure monitoring line was inserted through the right superior pulmonary vein before weaning from CPB support.

The perioperative scores of the patient were 4, 12, and Class II (adequate minor residua) in the Risk Adjustment in Congenital Heart Surgery (RACHS-1) score, the Aristotle Comprehensive Complexity (ACC) score, and the Technical Performance Score (TPS), respectively.

Due to insufficient LV contractions and hypotensive progress following weaning from CPB, the patient was switched to ECMO support at the postoperative 2^nd^ hour in the intensive care unit. Neck cannulation was performed. He was cannulated from the right carotid artery, for the arterial flow, and from the right atrium, for the venous flow. An additional 50 to 100 IU/kg of heparin was introduced according to the initial activated clotting time (ACT). Continued heparin infusion was initiated at a dosage to maintain the ACT at 180 to 200 seconds, and the dosage was titrated. The ECMO pump flow was initiated at 100 ml/kg/min. The pump flow was rearranged after the maintenance of end organ perfusion was ensured, along with the increase in systemic venous oxygen saturation (SO_2_) and decrease in lactic acidosis.

Inotropic support was administered as milrinone (0.5 µg/kg/min) and a low dose of epinephrine (0.05 µg/kg/min) for the first few postoperative hours. Noradrenaline treatment was added if the coronary perfusion pressure was less than 20 mmHg. Fentanyl and dexmedetomidine were used for sedation and analgesia.

Cardiac functions were evaluated daily by echocardiography. Mechanical ventilation was initiated for oxygenation and to prevent atelectasis. Generally, the frequency was set at 10 to 12 breaths/min, fraction inspired oxygen (FiO_2_) at 0.35 to 0.45, and positive end-expiratory pressure (PEEP) at 5 to 10 cmH_2_O to provide a peak inspiratory pressure (PIP) <20 cmH_2_O.

**Fig. 2:**
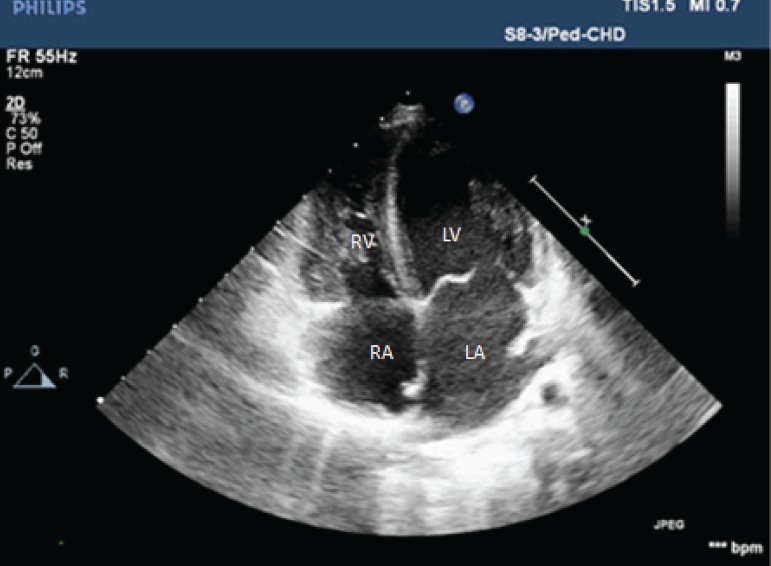
Postoperative 20^th^ day. Apical four-chamber transthoracic echocardiography. LA=left atrium; LV=left ventricle; RA=right atrium; RV=right ventricle

The patient was monitored with daily echocardiographic examinations, electrocardiography (ECG), and hemodynamic findings. The ECMO support was reduced gradually, and the patient was disconnected from it at the end of one week. On the 10^th^ day postoperatively, the general condition of the patient was good, and the LV had returned to the normal shape from the original banana shape (Figure 2). There was no systolic dysfunction, as confirmed by ECG. The pro B-type natriuretic peptide (BNP) level decreased from > 35,000 pg/L to 2,100 pg/L.

As the patient’s hemodynamic variables progressed to normal values, medical inotropic support was gradually reduced and discontinued completely. Echocardiographic examinations on the 16^th^ day postoperatively demonstrated normal systolic functions with mild insufficiency of the neo-aortic valve and a small left-to-right shunting through the opening left on the atrial septum. The patient was discharged without any neurological sequelae on the 20^th^ day postoperatively (Table 1).

**Table 1:** Timeline of the case's clinical and laboratory findings.

Day	ECMO	Short fraction (%)	Mitral regurgitation	BNP level	Mean vasoactive inotropic score	Treatment
2	1/2 flow	18	Severe	>35.000	21	Milrinone, epinephrine, noradrenaline
3	1/2 flow	18	Severe	>35.000	17	Noradrenaline ceased
4	1/2 flow	20-22	Severe	30.000	15	Lisinopril initiated
5	1/3 flow	24	Moderate	16.000	10	
6	1/4 flow	26	Moderate	9.700	7	Epinephrine ceased
7	ECMO weaning	28-30	Mild	2.100	5	
10		32	Mild		5	Extubation
15		32-34	Mild		0	Milrinone ceased, bed service transfer
20		32-34	Mild	1.150	-	Discharged

BNP=B-type natriuretic peptide; ECMO=extracorporeal membrane oxygenation

In the follow-up conducted one month after discharge, the patient was hospitalized again with a diagnosis of pneumonia. Echocardiography revealed moderate mitral and aortic regurgitation with a reduced contraction fraction (20%). The mean pulmonary artery pressure measured by pulmonary regurgitation was 50 mmHg. The patient was treated with antibiotics for three weeks. After treatment, sildenafil, enalapril, and furosemide were initiated due to ongoing pulmonary hypertension and heart failure findings. At the seven-month follow-up, it was observed that regurgitations were reduced, pulmonary artery pressure decreased, and cardiac function normalized (contraction fraction: 30 to 35%, ejection fraction: 60%). One year after the operation, the patient had no issues.

## DISCUSSION

The arterial switch procedure is a question of debate in patients with TGA/IVS after three weeks of age due to the LV structure and function loss. Recently, because of the potential medium- to long-term complications of these operative procedures, there has been an increase in reports of successfully performed arterial switch procedures after the 3^rd^ week of life with the aid of improvements in advanced life support systems^[4,5]^.

Sarris et al.^[6]^ reported on the collective experience of 19 European centers. Fifty-two patients with TGA/IVS who were older than four weeks of age (36 patients were older than eight weeks) underwent primary ASO with a mortality comparable to that of younger patients (2% *vs*. 3%). Yildiz et al.^[7]^ did not report any mortality in a series of 13 patients older than three weeks of age.

As the loss of muscular mass decreases the chance that the LV can function as the systemic ventricle as time goes by, confirming the suitability of primary ASO is one of the most important points in the evaluation of late TGA. Furthermore, the exact time of a complete loss of the opportunity to perform the operation is unknown. Echocardiography is an important tool for determining the type of surgery suitable for these patients. Straightness of the IVS, age-corrected thickness of the LV posterior wall, and LV myocardial mass (> 35 g/m^2^) are important factors in the decision^[7]^.

For the success of the primary ASO, the LV needs to adapt quickly to the high afterload postoperatively. Usually, an unfavorable LV geometry is accepted as a contraindication for primary ASO, but this is reported to be transient and can be treated by pharmacological means and ECMO support^[5,7]^. This change in LV shape is due to a low afterload, not an intrinsic change in the LV myocardial properties, and it is reversible^[5,7]^.

Although an increased need for ECMO support has been reported in late ASO patients, this was not accompanied by an increase in mortality. Bisoi et al.^[5]^ reported 20% of ECMO support and 3.7% mortality in a series of 109 late ASO patients. However, late mortality was reported separately from primary disease.

It has been reported that patients undergoing late ASO have prolonged hospital stays, more LV mechanical and ventilatory support, and delayed closure of the chest. However, a recent study found out that there were no statistically significant differences in the mean duration of ventilation, intensive care unit and hospital stays, inotropic agent need, mortality, diaphragm paralysis, acute renal failure, pericardial effusion, pleural effusion, arrhythmia, or chylothorax incidence^[4]^.

Recently, ECMO has been used for cardiac surgery patients in several situations, such as in cases of recurrent cardiac arrest requiring prolonged resuscitation, intractable hypotension despite inotropic support, resistant ventricular tachycardia and fibrillation episodes, myocarditis with low cardiac output despite medical treatment, and support required in the pre- and post-transplantation periods. Extracorporeal life support (ECLS) is not an innocent treatment; associated mechanical, hemorrhagic, renal, pulmonary, and endocrine complications have been reported in literature^[8]^. We did not observe any complications in our patient.

There are different types of surgeries performed in various complex congenital heart diseases. Therefore, scoring systems are needed to evaluate mortality and morbidity. RACHS-1, ACC, and TPS are three of these scoring systems. It was reported in many studies that these scoring systems were valuable in predicting mortality and morbidity^[9,10]^. And these three scoring systems were evaluated in our case report.

Patients should be followed up for complications requiring reintervention after late ASO, such as pulmonary anastomosis or peripheral stenosis, neo-aortic valve insufficiency, neo-aortic root dilation, coronary artery insufficiency, and disposition to atherosclerosis^[4]^. Although mild aortic insufficiency is frequent and rarely progresses to a clinically significant level following timely ASO performance, late performance of ventricular septal defect (VSD) closure and ASO are the most important risk factors for the development of aortic insufficiency^[4-6]^.

## CONCLUSION

In conclusion, late ASO can be performed safely in suitable patients with TGA/IVS. This should be done after a detailed evaluation and with efficient, advanced, and suitable postoperative intensive cardiac unit monitoring.

**Table:** 

Author's roles & responsibilities	
SH	Substantial contributions to the conception or design of the work; drafting the work or revising it critically for important intellectual content; final approval of the version to be published
EO	Drafting the work or revising it critically for important intellectual content; final approval of the version to be published
OY	Substantial contributions to the conception or design of the work; drafting the work or revising it critically for important intellectual content; final approval of the version to be published
BT	Substantial contributions to the conception or design of the work; drafting the work or revising it critically for important intellectual content; final approval of the version to be published
AG	Substantial contributions to the conception or design of the work; drafting the work or revising it critically for important intellectual content; final approval of the version to be published
